# Blockchain in Health Care Innovation: Literature Review and Case Study From a Business Ecosystem Perspective

**DOI:** 10.2196/19480

**Published:** 2020-08-31

**Authors:** Shuchih Ernest Chang, YiChian Chen

**Affiliations:** 1 Graduate Institute of Technology Management National Chung Hsing University Taichung Taiwan

**Keywords:** blockchain, health care industry, business ecosystem, smart contract, paradigm shift

## Abstract

**Background:**

Blockchain technology is leveraging its innovative potential in various sectors and its transformation of business-related processes has drawn much attention. Topics of research interest have focused on medical and health care applications, while research implications have generally concluded in system design, literature reviews, and case studies. However, a general overview and knowledge about the impact on the health care ecosystem is limited.

**Objective:**

This paper explores a potential paradigm shift and ecosystem evolution in health care utilizing blockchain technology.

**Methods:**

A literature review with a case study on a pioneering initiative was conducted. With a systematic life cycle analysis, this study sheds light on the evolutionary development of blockchain in health care scenarios and its interactive relationship among stakeholders.

**Results:**

Four stages—birth, expansion, leadership, and self-renewal or death—in the life cycle of the business ecosystem were explored to elucidate the evolving trajectories of blockchain-based health care implementation. Focused impacts on the traditional health care industry are highlighted within each stage to further support the potential health care paradigm shift in the future.

**Conclusions:**

This paper enriches the existing body of literature in this field by illustrating the potential of blockchain in fulfilling stakeholders’ needs and elucidating the phenomenon of coevolution within the health care ecosystem. Blockchain not only catalyzes the interactions among players but also facilitates the formation of the ecosystem life cycle. The collaborative network linked by blockchain may play a critical role on value creation, transfer, and sharing among the health care community. Future efforts may focus on empirical or case studies to validate the proposed evolution of the health care ecosystem.

## Introduction

### Background

In the last decade, blockchain technology has gained growing attention from both academia and practitioners in a range of industries, including banking, insurance, trade, and medicine. Blockchain has potential in various industries, including in financial applications, supply chains [1], the insurance industry [2], and even medical health care records [3-5]. Through maintaining an immutable, tamper-proof, consecutive list of transactional data in a distributed network, blockchain has created several disruptions in incumbent business processes with its unique features. Having a promising capability to improve information flow, sharing, and transmission among participating nodes (ie, partners in the real system), blockchain is expected to transform legacy operations with innovative service delivery and ownership transfer [6]. Blockchain adoption and pioneer pilots in different sectors have shown its power in transforming traditional working paradigms.

Blockchain, as a kind of distributed ledger technology, enables data storage, sharing, and verification under a distributed peer-to-peer network [7]. Participating nodes (ie, entities) may cooperatively maintain the common shared ledger by contributing efforts to data verification via cryptography. Blockchain can be viewed as a consecutive list of transactions that are chronologically appended to the previous ones. Updates of any part need to be verified and then recorded on the chain. This process is achieved by participating nodes’ contributions to solving the cryptographical puzzle, which in turn increases the difficulty of malicious tampering and alterations. In this sense, all transactions are visible and immutable for all parties, thus providing audit trails and data integrity. In addition, its affiliated technology, smart contracts, can be deployed on blockchain-based platforms to activate or enforce specific desired processes. Smart contracts are computer protocols that aim to execute terms of a contract or agreements [8]. In real practice, smart contracts can be coded with computer languages to interact with one another and be triggered by events in the real world [9]. These attributes, when deployed on blockchain system, may facilitate business logic and process automation.

**Figure 1:**
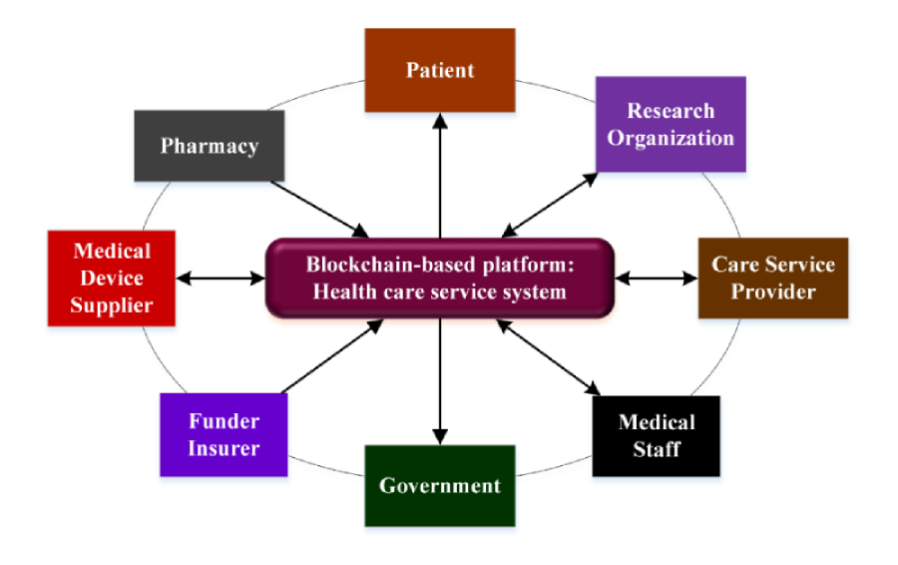
Typical health care ecosystem.

Recent publications, including technical reports, research articles [10,11], and consulting papers [12], have addressed blockchain’s potential to reshape the complex operations in the field of health care. Blockchain applications in the realm of health care may be promising; however, the compositions and interactions among major health care stakeholders, such as patients, care service providers, pharmacies, funders and insurers, medical device suppliers, and research organizations, are rather complex (see Figure 1). Extant research topics on how these stakeholders may achieve benefits by the use of blockchain technology have been addressed from the perspective of a single industry. Comprehensive discussions on the development and potential evolution of blockchain-based health care have been discussed less. It is noted that activities and interactions among stakeholders may have crossed a variety of industries. As Moore [13] has suggested, a careful systematic approach to business strategy needs to consider firms in the scope of a larger ecosystem rather than a member of a single industry. To better elucidate the evolution of a health care ecosystem utilizing blockchain innovation, stakeholders must address cooperative and competitive issues when attempting to deliver tangible and intangible values to meet customer needs.

Through unique distributed schemes and immutable shared ledgers, blockchain allows better transparency, security, privacy, traceability, and trust-free environment among players [14]. This implies that blockchain connects not only individual siloed databases via decentralized governance but also the ecosystem surrounding health care stakeholders. However, this may lead to more complex supply-and-demand relationships and interactions among actors who operate their businesses in an original centralized manner. Therefore, this study attempts to shed light on driving inertia from a *business ecosystem* perspective rather than through a traditional supply chain vision. Moore [15] defined the business ecosystem as an economic community loosely connected by a group of interacting organizations and individuals who share common values and who coevolve with one another. Researchers also extended this argument by addressing cross-industry collaboration rather than disparate interactions among directly connected counterparties [16,17]. This concept provides broader visions when blockchain interplays, connects, and disintermediates the dynamic relationships among connected medical communities, service providers, and end customers.

However, there is very limited research on blockchain-based health care ecosystems in the extant literature. Previous research efforts on blockchain mainly focused on technological potential [18], individual applications [19], medical record accessibility [20], and general influence. Others discussed the proof-of-concept of system design [21,22], adoption attitudes [23], governance, challenges, and opportunities in future research [24-26]. Few extant articles in the literature have addressed dynamic relationships among medical stakeholders with an overview of the blockchain ecosystem. Therefore, this research aims to investigate how blockchain can lead to a coevolving health care ecosystem by collating overviews of potential evolutions of blockchain-enabled health care applications from recent literature from a perspective of the business ecosystem. In this study, we address two research questions:

1. Research question 1: What kind of potential effects from recent innovations and applications make use of blockchain in the health care industry?
2. Research question 2: How do health care stakeholders participate, interact, and evolve in the blockchain-based ecosystem and how do they collaboratively contribute to a potential paradigm shift?

To shed light on blockchain’s influence on value creation and capture of medical stakeholders, we examine and address these research questions from a perspective of the business ecosystem, with an aim to contribute to the body of knowledge in health care.

### Existing Service Process and Blockchain Roles

**Figure 2:**
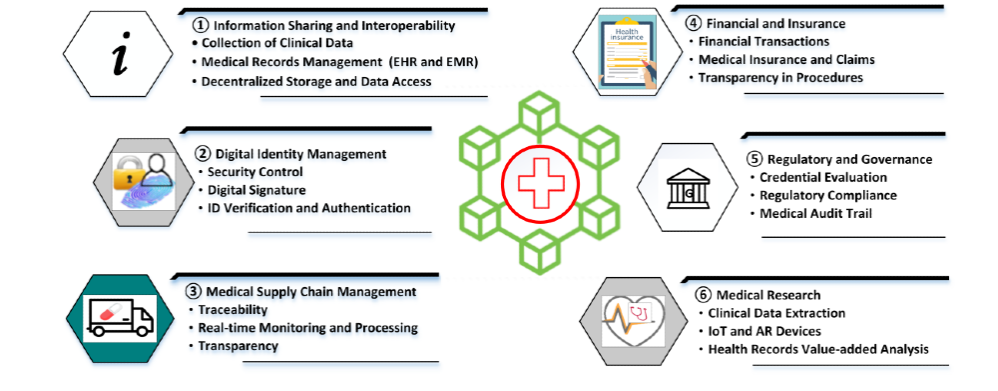
Blockchain’s role in improving the health care service system. AR: augmented reality; EHR: electronic health record; EMR: electronic medical record; IoT: internet of things.

Traditionally, medical information is located at disconnected databases in clinics, labs, or medical institutions. Aggregating health data from disparate sources and gaining a holistic view of patient treatment history have been difficult and costly. As blockchain can store transaction logs among participants, better transparency and completeness of treatment history could be achieved. Blockchain may drive the digital transformation of legacy information sharing [27]. Traditional paper-based processes and manual processing could be reduced and better interoperability among disconnected health systems is feasible. In addition, traditional medical supply chains have suffered from poor traceability and invisible provenance. Blockchain may provide solutions to improve transparency and real-time monitoring from manufacturing to delivery. Other focused areas also include secure identity management [28], audit and governance, and facilitation for medical research (see Figure 2).

## Methods

### Literature Review

To answer research question 1, we conducted a literature survey to find the current state and potential of blockchain applications in the health care field. Other than using a systematic approach, we focused on specific applications that may be enabled by blockchain to transform the interaction and manipulation of a health care ecosystem. Some review articles in the recently published literature were also selected to help understand the potential evolution among health care stakeholders.

**Figure 3:**
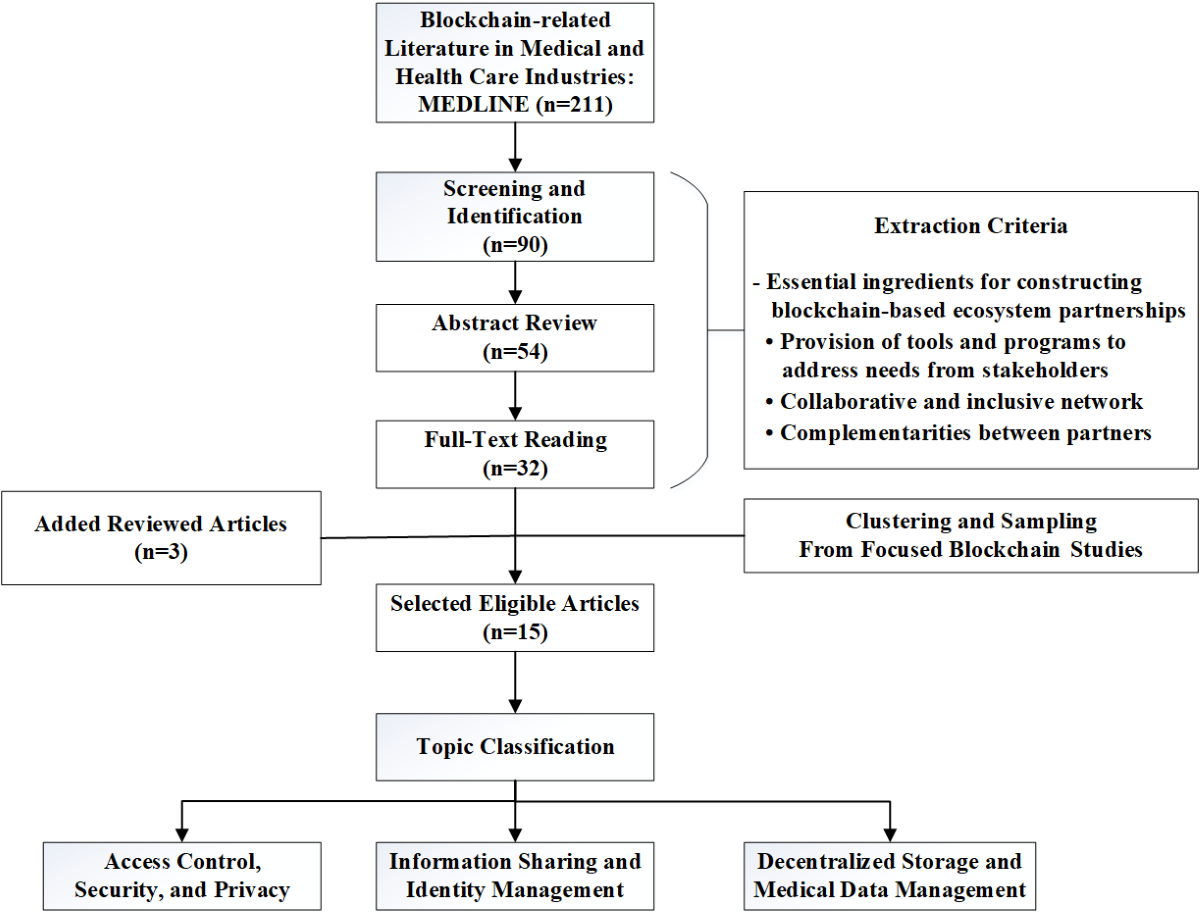
The procedural framework for the focused literature review. MEDLINE: Medical Literature Analysis and Retrieval System Online.

Figure 3 illustrates the search and review process of the focused literature. We searched for blockchain studies in medical and health care fields and conducted subsequent article screening and identification; abstract and text reviewing were conducted to select focused literature. The numbers in the flowchart boxes in Figure 3 denote articles that were available after the respective procedural steps. From the ecosystem perspective, the literature selection and extraction criteria paid attention to the capabilities that relevant studies highlight and that elucidate essential ingredients for constructing blockchain-based ecosystem partnerships. Some review articles were added to give a general overview of blockchain-based health care studies. Sampled articles were extracted from the filtered corpus to highlight focused topics, such as data management, information sharing, access control, security, and privacy.

### Case Study

In this study, recent blockchain-based health care projects were examined to shed light on the disruption to health care practice. The case study, a qualitative method, refers to a systematic analysis of a specific target from a wide perspective and enables comprehension throughout the exploration process [29]. Applying this approach, researchers suggested that consideration of research objectives, contexts, and representativeness needs to be stressed [30,31], followed by inclusion of selection protocols suggested by previous literature articles, secondary resources from news archives, consulting reports, company websites, and academic articles [32]. Case study results were then collated to answer research question 2 and to elucidate the understanding of cooperative and competitive strategies and operational business schemes in the health care context. We selected the IBM blockchain–health care initiative [33] as the body of the target case and combined the concept of the business ecosystem with the health care context to analyze the interactions, cooperative or competitive, among species (ie, ecosystem members). Furthermore, major players’ roles and influences in the blockchain-based health care ecosystem were analyzed to give research implications.

### Business Ecosystem Perspective

#### Overview

This study analyzed the potential evolutionary path of blockchain-based health care innovation from a business ecosystem perspective. Moore [13] proposed the life cycle of a business ecosystem and divided it into four stages: birth, expansion, leadership, and self-renewal or death. We identified four major development stages within which health care stakeholders interact with each other and evolve chronologically with their roles and cooperative and competitive challenges. Iansiti and Levien [34,35] extended Moore’s concepts by defining the roles of actors and argued that these roles were formed by large, loosely connected networks of entities. They further classified three categories of the actors’ roles as keystone, dominator, and niche player. The business ecosystem is comprised of diverse participants across various industries. The overall health of the ecosystem depends on the positive interactions and operations among stakeholders.

#### Birth: Pioneering

During this stage, entrepreneurs focus on the value creation or proposition that meets customers’ needs. The product or service needs to be presented in its best form to draw potential customers’ attention and effectively deliver its value. Leaders in the ecosystem aggregate suitable suppliers to take part in the environment and attempt to incorporate business partners’ capabilities to optimize the value package to customers.

#### Expansion

The ecosystem grows and expands its territories. The business ecosystem faces competition to increase market share against its rivals. Firms may devote a large number of endeavors to marketing activities for increasing sales. Meanwhile, to improve overall performance, issues regarding large-scale adoption and distribution are stressed. In this stage, while incomplete ecosystems are likely to be expelled from competition, superior ones may integrate community members to complete sound supply chains, thus achieving ecosystem stability. Required conditions in the expansion stage include value-oriented business concepts and the corresponding potential to broaden scalability.

#### Leadership: Authority

Following the expansion, the leader or integrator needs to guide the direction of investment and technology standards. As innovation is a crucial factor for evolving ecosystems, stakeholders may find their positions and revenue models through the leader’s guidance. While the bargaining power of suppliers increases during this stage, the system integrator needs to enhance the supply chain management with alternative options to assure the stability of production and distribution. How firms constantly create values to maintain their importance in the ecosystem is critical to the overall health and continued improvement of the ecosystem.

#### Self-Renewal or Death

This stage occurs when firms face external threats, for example, changes in regulations or the rise of new ecosystems and innovations such as emerging technologies. Original business communities may undergo different levels of change and fluctuation. The altered environment may challenge the survival of original members. How leaders detect the potential changes and new incoming elements and threats, thereby correspondingly reacting to these alterations, may decide the future outcome of the ecosystem. When facing obsolescence, either self-renewal by incorporating new innovative ideas or stepping toward death depends on the capabilities to enable system transformations.

## Results

### Reviewed Literature

#### Overview

The pursuit of building a sustainable and healthy ecosystem is essential for participating stakeholders. When the requirements for building a health care ecosystem are considered, we found issues that are being addressed by extant research studies. We selected a number of articles to elucidate the recent research foci. Table 1 summarizes several related articles regarding blockchain in health care; these articles were published in academically rigorous peer-reviewed scientific journals. Focused topics in the blockchain–health care ecosystem are briefly collated in the following sections.

#### Decentralized Storage and Medical Data Management

Centralization of health data from disparate sources has long been a major pain point for further medical usage. Generally, disconnected data sources could be utilized to increase the integration and aggregation of medical data. Based on the distributed nature of blockchain, researchers have stressed that there are data storage and management issues in clinical trials [22], insurance [2], and personal health scenarios [36].

#### Information Sharing and Identity Management

Based on the immutable and distributed features of blockchain, a common shared ledger may facilitate health information exchange (HIE). Some proof-of-concept studies have covered the potential and major contributions to these topics; for example, Ali et al [37] focused on remote health monitoring, Hau et al [23] surveyed stakeholders’ attitudes, and Esmaeilzadeh and Mirzaei [18] conducted an experimental study to understand patients’ perceptions of various exchange mechanisms. In addition, while several researchers conducted literature reviews to shed light on potential strengths and limitations of blockchain applications [38,39], others reviewed potential identity management solutions [28] and developed evaluation frameworks for assessing performance of blockchain initiatives [40].

#### Access Control, Security, and Privacy

As access control and authentication are major security requirements for managing health care and medical data, researchers have proposed some blockchain-based prototypes to provide solutions to current health systems [41,42]. Digitization of electronic medical records (EMRs) may introduce cyberattack risks on data security and privacy when stakeholders, such as providers, payers, and researchers, attempt to interact with patient data. Blockchain-enabled solutions may maintain patient-sensitive data through a friendly approach [43-45].

**Table 1:** Overview of blockchain-based health care applications in the research literature.

Article, author (year)	Research method	Focus	Key findings
Maslove et al (2018) [22]	Proof-of-concept	Clinical trials data management	A web-based interface, BlockTrial, allowed patients to grant researchers access to their data and allowed researchers to submit queries for data that are stored off-chain. The proposed system increased the trustworthiness of data collected during clinical research, with benefits to researchers, regulators, and drug companies alike.
Zhou et al (2018) [2]	Proof-of-concept	Medical insurance storage system	A blockchain-based medical insurance system, MIStore, deployed on Ethereum was proposed to serve as a medium for accountable record keeping. Medical insurance data were better managed in a distributed way.
Chen et al (2018) [36]	System design	Secure medical data storage and service framework	A storage scheme to manage personal medical data based on blockchain and cloud storage was proposed without third parties. No single party would have absolute power to affect the processing; better and more secure medical storage could be achieved.
Ali et al (2020) [37]	Proof-of-concept	Remote health monitoring and data sharing	A solution for patients to share biomedical data with their doctors was proposed without manipulation by trusted third parties. In various health monitoring scenarios, three use cases—cardiac monitoring, sleep apnea testing, and electroencephalogram following epileptic seizures—were tested for system feasibility.
Hau et al (2019) [23]	Survey	Attitudes on information sharing	Medical doctors reported significantly more negative attitudes than patients. Furthermore, self-employed doctors reported more negative attitudes than employed doctors and university professors.
Esmaeilzadeh and Mirzaei (2019) [18]	Experimental study	Medical information exchange	Significant differences existed in patients’ perceptions of various exchange mechanisms with regard to patient privacy concern, trust in competency and integrity, opt-in intention, and willingness to share information. Participants held a favorable attitude toward the implementation of blockchain-based exchange mechanisms for privacy protection, coordination, and information exchange purposes. This study proposed the potential strengths and limitations of a blockchain-based attempt within a health information exchange context.
McGhin et al (2019) [38]	Literature survey and case study	Research challenges and opportunities	The survey presented a careful examination of specific blockchain issues and how they affect the health care industry. Health care industry requirements and blockchain potential effects in supporting these requirements were discussed.
Vazirani et al (2019) [39]	Systematic review	Blockchain implementation	Of the 71 included studies, the majority discussed potential benefits and limitations without evaluation of their effectiveness, although some systems were tested on live data.
Bouras et al (2020) [28]	Literature review	Identity management	This study presented state-of-art decentralized identity management using blockchain and highlighted the possible opportunities for future adoption. Decentralized models and pilot projects were presented to give implications.
Zhang et al (2019) [40]	Framework construction	Development of balanced scorecard evaluation framework	A framework was proposed to holistically assess the performance of blockchain initiatives in providing value-based care. By extending the concept of existing balanced scorecard evaluation, both the financial and nonfinancial benefits of blockchain initiatives were evaluated.
Shuaib et al (2019) [41]	Literature review	Blockchain potential in improving secured digitized medicine	The digital ledger technology could be used to improve current systems. Data are distributed and decentralized, preventing loss and allowing recovery in the event of an attack. Audit trails keep track of what transactions and modifications are made to patient records, while notifying all users on the network. Patients will be given more control over who has access to their data by selecting who carries the cryptographic keys required to decrypt and view them. In addition, issues such as scalability need more research efforts.
Guo et al (2018) [42]	System design	Secure signature authentication	An attribute-based signature scheme with multiple authorities, in which a patient endorses a message according to the attribute while disclosing no information other than the attested evidence, was proposed. By sharing the secret pseudorandom function seeds among authorities, this protocol resists collusion attack out of N from N–1 corrupt authorities.
Kadam et al (2019) [43]	System design	Patient data privacy	Patient data were secured by applying the Secure Hash Algorithm for the generation of hash values and the Paillier algorithm to re-encrypt the same information regarding patient data that is divided among a number of different servers. This approach increases the difficulty of hacker access and attack and maintains the security principles (ie, availability, integrity, and confidentiality).
Al Omar et al (2019) [44]	System design	Health care data privacy	A patient-centric health care data management system was proposed by using blockchain technology for storage, which helped to attain privacy. Cryptographic functions were used to encrypt patients’ data and to ensure pseudonymity.
Yue et al (2016) [45]	System design	Health care data privacy	The Healthcare Data Gateway architecture, using blockchain, enabled patient-centric data management (ie, own, control, and share patient data) in a secure way without violating privacy, which improves the intelligence of health care systems. The proposed access model ensures better manipulation of health care data and enables untrusted third parties to conduct computation with patient data without violating privacy.

### Case Study of the IBM Blockchain–Health Care Initiative

On January 24, 2019, IBM announced its collaborative blockchain initiative with major health care players, including Aetna (acquired by pharmacy and health plan provider CVS Health), Anthem (health plan provider), Health Care Service Corporation (the largest customer-owned health insurance provider in the United States), and PNC Bank [46]. IBM has been searching for new opportunities by leveraging the potential of blockchain and attempting to build up a special networked health care ecosystem. In the last few months, health organizations, health care providers, start-ups, and technology companies joined in this initiative to grow the Health Utility Network, of which Cigna and Sentara Healthcare are participants. The aim is to drive digital transformation by providing better transparency and interoperability. Participants may reap benefits from building, sharing, and deploying solutions to incumbent challenges in the health care context. Major issues and potential blockchain use cases are enumerated as follows:

1. Provenance and traceability of pharmaceutical supply chain: fake and counterfeit drugs could be troublesome and dangerous issues as drug provenance is difficult to track in a cross-border setting. A large number of handovers from manufacturers, shippers, distributors, retailers, and pharmacies may cause inaccuracies and disputes in medical delivery operations. Counterfeit drugs with improper ingredients and dosages may jeopardize the health of patients and even cause legal disputes among manufacturers, suppliers, and customers. With immutable, tamper-proof, and trackable characteristics, blockchain may provide solutions to authenticity and traceability of transferred assets along with auditable and secure transaction records among stakeholders. For example, in a private drug blockchain, drug registration by pharmaceutical companies may grant a higher level of trustworthiness and authentic proof. Also, these companies, acting as dominators, could assign the roles of the actors; some of them may have the rights for registration while others may conduct verification of transactions. The provenance of drugs can be assured via verification processes with related manufacturing or identity information when appended on-chain, making it easy to be tracked.
2. Data management during clinical trials: when clinical trials are implemented, numerous data are produced by different devices via the operation of medical staff. How these data are stored, transmitted, shared, and utilized for medical therapy or operations is critical to existing manual systems. Errors and fraud during clinical trials operations could be generated via malicious alterations or unintentional mistakes. Typical flaws could occur when trial procedures are inaccurately designed by biased intentions from actors or inconsistent records and responses from patients’ evolutionary medical reports. Blockchain in this case could provide proof-of-existence for any form of documentation. The information needs to be verified via the consent of the participating nodes and not under a single entity’s control. Modifying or changing information would be cryptographically difficult to conduct among a majority of network players, thus making documentation highly trusted.
3. EMR and electronic health record (EHR) management: where patient or medical records are concerned, a challenge is that individual medical data are not easily accessed by different medical institutions or clinics. While the medical information is stored disparately in various databases or systems, it is difficult to deliver proper medication and care service in a personalized context. Sensitive data can also hinder the transmission efficiency among medical organizations. How to access, share, and utilize a holistic medical treatment history in a secure way remains a challenging issue in centralized EMR systems. However, with the help of distributed ledger technology, blockchain may have potential regarding the manipulation and access control of such EHR and EMR systems. Blockchain platforms can be combined with existing EHR and EMR systems, either in the cloud computing environment or otherwise, through the use of Oracle and data gateways. Patients can share their medical records, with or without permission, to registered users or stakeholders on a medical blockchain. Patients may decide the level of information disclosure through smart contract settings to specific users, thus receiving rewards from the blockchain system, accordingly. As described above, blockchain could facilitate the sharing and management of EHRs and EMRs among supply and demand entities. Related data analysis and rewards from sharing could potentially promote the participation of the medical community and, consequently, leverage a network effect.

In health care, major inefficiencies can arise from clinical operations, administrative processing, and frictions among disparate systems. These pain points have decreased the overall performance and have led to poor customer experiences in regard to incumbent medical and health care systems and services. The act of incorporating major players through blockchain-based systems and services in health care may help to develop a healthy, open-networked, and collaborative ecosystem. The blockchain-enabled collaboration aims to address the aforementioned challenges by pursuing reduced administrative error, mitigated system frictions, streamlined claims and payment transactions, and efficient information exchange. Iansiti and Levien expanded Moore’s ecosystem view and proposed the strategies that firms might adopt to position themselves in the business ecosystem. The strategic roles include keystone, niche player, and physical dominator. The keystone in the business ecosystem provides a platform to which niche players add value and build offerings. Niche players account for the bulk proportion of the ecosystem and are responsible for value creation and innovation. The physical dominator directly controls the majority of a network via horizontal or vertical integration. In an IBM blockchain ecosystem, the major players’ roles and corresponding functions are shown in Table 2 and are summarized as follows:

1. IBM: keystone—blockchain platform provider and coordinator.
2. Aetna of CVS: niche player—improves data accuracy and optimization of health care system operation.
3. Anthem: niche player—medical information exchange.
4. Health Care Service Corporation: physical dominator—reduces information fragmentation and improves claims procedures and health care system connection.
5. PNC Bank: niche player—facilitates payment transactions and supports medical finance.

**Table 2:** Major players’ roles and influences in a blockchain-based health care ecosystem.

Major player type	Major players	Roles	Influences on the ecosystem
Keystone	IBM	Platform provider in the ecosystem Aim to create opportunities for niche players and support the operation of the whole system	Enable the establishment of a healthy environment, which leads to an organization’s survival and prosperity Convene followers to achieve diversity
Physical dominator	Health Care Service Corporation or other health care service providers	Integrators in the ecosystem Integrators directly own and manage a large proportion of a network by using vertical or horizontal measures	Provide most products and services to meet customers’ needs Exploit their positions to take over the network and extract the created value
Niche player	Aetna of CVS, Anthem, or PNC Bank	Value creators and innovators in the ecosystem Focus all potential endeavors on enhancing their narrow domain of expertise	Leverage complementary resources from others to create differentiated value Competition and cooperation of niche players support the coevolution of the ecosystem

Embracing of blockchain technology is not the privilege of this initiative only. Competitors making similar efforts, such as Change Healthcare, Hashed Health, Guardtime, Gem, and SimplyVital Health, have also teamed up to launch a blockchain pilot—Intelligent Healthcare Network with Blockchain Processes—in the realm of health care. Other competing projects with a more-or-less different focus have also led to consortia competition. Prominent examples include Synaptic Health Alliance, targeting provider directories and data reconciliation, and ProCredEx, focusing on storage and sharing of medical credentials. PNC Bank, acting as a partner of interdisciplinary alliance, stands in a public position and contributes its edge to facilitate transactions among patients, payers, and providers in both domestic and cross-border contexts.

### Business Ecosystem With Evolutionary Life Cycle

Blockchain, as an emerging technological innovation, has provided opportunities for incumbent health care stakeholders. As for the IBM case, a collaboration of health care partners has resulted in a new ecosystem. Its potential evolutionary stages have formed a business ecosystem lens; these stages are summarized in Table 3.

At the *birth* stage, the IBM blockchain–health care pilot faces consortia competitions from other allies. Even though the focused markets might be slightly different from pilot to pilot, similar efforts and common objectives for driving digital transformation in the health care industry are the same. IBM, as a recognized leading enterprise blockchain provider, possesses an advantageous edge against competitors. When entering into the *expansion* stage, the key focus is to bring new innovations to market to increase the market share. This could be carried out by optimizing platform functionality, absorbing complementary health care members, and addressing the changing demands for customers. In addition, to outperform rival ecosystems, it is essential to build up technical or industrial standards in terms of competitive strategy [47]. During the *leadership* stage, the leading ecosystem may focus on future prospects for followers. This could be implemented by compelling suppliers and customers to complete sound visions; for example, integration with other disruptive technologies, such as machine learning, artificial intelligence, mobile and ubiquitous health, wearables, and internet of things (IoT). Inversely, to prevent pressure from increased bargaining power, actions such as using backward integration, searching multiple suppliers, increasing profile, and conducting market education are needed. At the last stage, the blockchain–health care ecosystem may step toward *self-renewal or death*. This may depend highly on capabilities that the existing ecosystem may possess; it can either innovate or be replaced with alternative ecosystems or paradigms.

**Table 3:** The evolutionary path of a blockchain–health care ecosystem: the IBM case.

Stage	Cooperative challenges	Competitive challenges
Birth	Stakeholders create new value propositions of blockchain-based ecosystems and define their roles when working with suppliers and customers Players seize opportunities Example: IBM blockchain–health care ecosystem	Protect ideas against competitors with similar offerings Pilot cases with similar features Examples: Change Healthcare’s Intelligent Healthcare Network with blockchain processes, Synaptic Health Alliance, and ProCredEx
Expansion	Bring new innovations (ie, products or services) to market to increase the market share or coverage Strategy: optimize platform functionality, absorb complementary health care members, and identify and address changing demands from customers	Compete with and defeat rival implementations Expand market share by establishing market or technical standards Strategy: build up technical or industrial standards and expand the adoption of blockchain-based applications
Leadership	Make future prospects and encourage partners to step forward Measure: integrate with other disrupting technologies (eg, machine learning, artificial intelligence, mobile and ubiquitous health, wearables, and internet of things)	Maintain bargaining power against ecosystem players Measures: keep customers satisfied and strengthen the customer relationship management; use backward integration, search multiple suppliers, increase profile, and conduct market education
Self-renewal or death	Cope with innovators to generate or seize new opportunities or be replaced by alternative paradigms	Build high levels of entry barriers and customer switching costs to prevent being replaced by alternative ecosystems

## Discussion

### Comparative Analysis of the Existing System and the Future Ecosystem

Blockchain applications in the health sector have been receiving increased attention and prospects. We have summarized the current health care service pain points and highlighted the potential of blockchain in reshaping traditional practice and operations. Researchers have conducted literature reviews to report on the current challenges [48,49]. The major issues with the corresponding potential effects of blockchain are listed in Table 4.

**Table 4:** Health care service pain points and the potential effects of blockchain in the health care ecosystem.

Issue	Health care service pain points	Potential effects of blockchain leverage
Medical data storage	Highly disparate data sources across individual clinics or health care–related institutions	Decentralized data storage allows duplicate and immutable health records in the health network
Fraud and authenticity	Malicious attempts or human processing errors may cause fraud, alterations, or medical disputes Authorities are required for trust building among stakeholders Major issues include drug counterfeiting and provenance	Keeping critical items (ie, medical transactions or records) on blocks and permanently recording operations on-chain Mitigating the tampering issue via the verification and consensus architecture
Document type	Paper-based and manual processing causes difficulties in data aggregation	Supporting digitalized health documents deployed on secured shared ledger
Interoperability	Siloed data structures hinder interoperations across different databases	Blockchain-based networks enable interactions among health care stakeholders
Health claims and transactions	Inefficiencies that exist in clinical and administrative procedures and frictions among respective health systems have caused poor operations	Process automation facilitated by blockchain-based smart contracts enables streamlined claims and transaction procedures
Research data access and monetization	Challenges in aggregating, recruiting, and retaining data among medical parties and difficulties in monetization	Enabling of clinical trial data sharing and value-added analysis to create data use and monetization
Information sharing and transmission	Manual processing increases operational costs and expenditures Vulnerability and uncertainties from cyberattacks or system malfunction	Blockchain’s distributed attributes allow shared information in the health care network Consensus mechanism with tamper-proof features could reduce security and privacy concerns
Medical supply chain traceability	Uncertainties during handovers among participating parties Poor control in tracking user identities, ownership, and delivery status	Common shared ledger system allows for better transparency and monitoring on supply chain traceability Smart contracts can facilitate notifications of state changes

### Blockchain Impacts and the Changing Paradigm on the Health Care Ecosystem

#### Overview

This study collated blockchain-related literature in the health care industry. While many research efforts highlighted the potential effects of blockchain from a viewpoint of a single firm or industry, we attempted to shed light on its power from a more holistic manner, which focuses on the inclusive health care ecosystem. This changing and evolving paradigm may go through complicated cooperative and competitive challenges with the participating stakeholders. Therefore, from the illustrative case—the IBM blockchain–health care initiative—we elucidated and discussed the potential impacts and complex interactions during the lifecycle of component species or players. Five critical issues, when coevolving with blockchain adoption, are discussed to provide implications for researchers and practitioners.

#### Health Information Exchange With Interoperability and Integrity

HIE has long been a critical issue when data interoperability is considered; only with an effective information exchange scheme could the true value of health care information be unleashed [50]. Recently, a proliferation of publications and pilots have addressed the issue of medical records and health records. A decentralized scheme using a commonly shared ledger for information sharing offers innovators opportunities to disrupt traditional practice [51]. Health care data has granted blockchain-enabled applications great penetration points into the health care industry. Blockchain-enabled health care information exchange may unleash the power of blockchain to reduce frictions among siloed databases as well the costs from intermediaries [12]. To facilitate information exchange among disparate data systems across individual organizations, the transmission protocols or standards need to be addressed to provide data integrity. In so doing, an important part is the integration of transmission protocols, which mitigates effects of potential missing information and avoids incompatible situations. In addition, blockchain’s distributed framework may support cross-system health information usage. However, due to current technological limitations in designing blockchain applications, limited block size could become an issue for extended scalability. Therefore, only critical transactions will be appended on-chain and supporting data access schemes will be necessary for data manipulation. While blockchain could allow interoperability among health systems, incentives for individual stakeholders may become essential when creating beneficial models and supporting sustainable ecosystems are considered. In this regard, blockchain may unlock the true value of interoperability and achieve a higher level of disintermediation.

#### Digital Identity Management

Traditional identity management has been subject to the limitations of a centralized mechanism, such as security, privacy, and scalability. Centralized identity management is vulnerable to malicious attacks and alterations, thus being prone to theft, counterfeit, and fraud risks [28]. In addition, credentials required to request registration or access to health care services are also prone to misuse or to causing privacy disclosure. Distributed identity management may provide solutions to these limitations with its capabilities of ensuring data integrity and information sharing across different health care systems if deployed on an immutable and distributed network. The distributed model may also solve the duplicate and multi-version identity issues in health care use cases. Due to these features, identity owners may have full control of their unique digital identities and, in turn, enjoy benefits as the stakeholders in a valuable health care ecosystem. This implies that users have become the owners of their health data without the intermediation supported by traditional identity management systems. A higher degree of freedom to access, release, or share medical records has become possible. The blockchain-enabled digital identity is also useful for managing health care supply chain activities, such as the ownership transfer of specific assets. After all, as health care data are normally sensitive and confidential in nature, blockchain identity may leverage its characteristics to grant better security and privacy by reducing manual intervention and operational failures.

#### Health Care Supply Chain Management

Blockchain’s immutable and tamper-proof attributes have granted disruptive innovation to supply chain management. In a health care ecosystem, records of goods, such as drugs, and service flows could be recorded on-chain to provide better logistics visibility and timeliness. The integration of blockchain and medical IoT devices may be the next evolution of blockchain technology in the realm of supply chain management. A large amount of medical data generated by medical devices may be stored across different stakeholder systems. With the aid of blockchain, patient-generated data can be stored off-chain but accessed with permissions preset by blockchain-based smart contracts. In this regard, HIE can become more streamlined without intermediation. Another blockchain use case is for the drug or pharmaceutical supply chain. Typical pain points may occur during handovers across stakeholders. Blockchain provides better transparency on supply chain activities and players may have better control over product and service flows. Moreover, primary concerns also come from the provenance of drug supply. Serious fake and counterfeit drugs have prevailed due to poor authentication and traceability from manufacturing and shipping to delivery. The movement of drugs could be recorded on blockchain to provide better real-time monitoring as well as to cease the distribution of fake drugs. This implies that trackable footprints verified by participating players can help secure drug supply chains.

#### Medical Research and Data Exploitation

Medical records have long been managed with a centralized approach. However, the disconnected health systems that exist across different clinics or health organizations may hinder further usage of EHRs and EMRs for medical researchers [51]. A considerable number of medical records are stored in paper-based documents or in electronic health systems with poor interoperability. Poor efficiency in health care information exchange and rising costs of administrative processing have locked the true value of medical information. In traditional circumstances, researchers may have difficulties in acquiring patient data and medical records. This phenomenon may result from *where* and *how* questions. To address data sharing and exploitation among parties and research institutes, researchers have proposed a privacy-preserving model [52,53] and an incentive mechanism [54] during the course of data collection, sharing, and collaborative exploitation. With a shared health care ledger system, researchers may reap benefits from the blockchain-changing paradigm. They may access related data by checking smart contract conditions if the use is permitted by patients. Patients could get rewards or credits from the contributions or payments from researchers by granting different levels of permission, which are coded and stored by smart contracts on blockchain, to release specific data. In sum, blockchain may give control of data access to patients, and researchers could pay for access. In this regard, the traditional pain points for collecting patient data could be resolved in order to facilitate research conduct. Data reconciliation during research design and clinical trials may become easier with a shared medical ledger, thus improving health care and medical treatment.

#### Automation of Financial Transactions and Insurance Procedures

A lack of trust between health care stakeholders may affect the overall performance of financial transactions in the health care industry, for example, impedance in promoting alternative payment models between payers and providers. When the current reimbursement models and claim procedures were examined, we found hindrances on processing efficiency, transparency, and visibility among ecosystem members. For example, in current insurance fields, multiple middlemen and intermediaries exist throughout the procedures of health insurance policies. In addition, shared information could help insurers seek out better providers and provide verification on the fact if providers meet obligations and contractual terms. Smart contracts may replace efforts on drafting complex and value-based paper contracts and may automate the process of execution of terms or agreements. Through the aid of smart contracts, entities may set up logical process flows when preset conditions regarding health care activities are met. The deployment of smart contracts on decentralized immutable ledger systems could also make payment and claims records visible and render postaudit and review. In this sense, the paramount manipulation on data exchanges and payment transfer between insurers and their stakeholders could become easier and less expensive.

### Limitations

In this study, we conducted a literature review to investigate the potential impacts of blockchain-based health care innovations. Along with selected pilot cases, we discussed the positions and promises that blockchain may bring to the health care ecosystem. While researchers and practitioners have high hopes, challenges will be faced before the large-scale adoption of blockchain due to limitations from technical health care service operations and regulatory concerns. Confined by the level of blockchain maturity in various health care subsectors, different use cases and clinical trials need more support from empirical work to report on its real performance. We collated extant research efforts and attempted to shed light on a potential paradigm shift in the future health care ecosystem. Such an endeavor may be subject to uncertainties from the changing environment, technology limitations, or emerging innovations.

### Conclusions

This study aims to answer questions on the evolution and development of blockchain technology in health care research and on how stakeholders coevolve in this environment. From the perspective of the business ecosystem, we identified research articles about blockchain-enabled health care and we covered prototype designs and leading pilot cases in recent years. The evolutionary trajectory and interactions among major health care stakeholders may potentially formulate the blockchain-based health care ecosystem. Key players have presented their roles and interacted with one another to go through the life cycle of the business ecosystem. We illustrated their potential and the phenomenon of coevolution within the health care ecosystem. It is noted that while the literature in this field has proliferated recently, mostly regarding proof-of-concept studies, framework propositions, and trial pilots, a careful consideration on embracing such technology still needs to address technical limitations, privacy, mindset, and legal concerns. Our perspective and analysis show that large-scale adoption would need long-term support from health care stakeholders. Future research may devote more efforts to building up evaluation models to provide practical implications for practitioners. Whether feasible business models may sustainably survive in such an ecosystem needs attention from scholars. With a better understanding of how stakeholders coevolve within the ecosystem, players may reap their benefits in a more efficient manner to propel a potential blockchain–health care paradigm shift.

## Abbreviations

EHR: electronic health record
EMR: electronic medical record
HIE: health information exchange
IoT: internet of things
